# Anxiety Symptom Trajectories Predict Depression Symptom Trajectories up to Four Years After CBT for Youth Anxiety Disorders

**DOI:** 10.1007/s10802-024-01214-9

**Published:** 2024-06-15

**Authors:** Krister W. Fjermestad, Fredrik Ø. Norum, Helene S. Brask, Arne Kodal, Wendy K. Silverman, Einar R. Heiervang, Gro Janne Wergeland

**Affiliations:** 1 Department of Psychology, University of Oslo, Forskningsveien 3a, N-0317 Oslo, Norway; 2 Frambu Resource Centre for Rare Disorders, Siggerud, Norway; 3 Haukeland University Hospital, Bergen, Norway; 4 School of Medicine, Yale University, New Haven, USA; 5 Innlandet Hospital Trust, Lillehammer, Norway; 6 Faculty of Medicine, University of Bergen, Bergen, Norway

**Keywords:** Anxiety, Depression, Cognitive behavioral therapy, Longitudinal effects, Children, Parents

## Abstract

Long-term data on depression symptoms after cognitive behavioral therapy (CBT) for youth anxiety disorders are scant. We examined depression symptoms up to four years post CBT for anxiety addressing youth age and gender, family social class, and parent mental health as predictors. The sample comprised 179 youth (*M* age at pre-treatment = 11.5 years; *SD* = 2.1) in a randomized controlled trial. Clinically assessed anxiety diagnoses and youth and parent-reported anxiety and depression symptoms were measured before, after, and one and four years after CBT. Parent self-reported mental health was measured before CBT. We used regression analyses to determine whether full diagnostic recovery at post-CBT predicted depression trajectories across the four-year assessment period. We used growth curve models to determine whether anxiety trajectories predicted depression trajectories across the four-year assessment period. Youth who lost their anxiety diagnoses after CBT had significantly lower parent-reported depression levels over time, but not lower youth self-reported depression levels. The anxiety symptom trajectory predicted the depression symptom trajectory up to four years post-treatment. There was more explained variance for within-informant (youth-youth; parent-parent) than cross-informants. Being older, female, having lower socio-economic status and parents with poorer mental health were associated with more youth-rated depression over time. However, these demographic predictors were not significant when anxiety symptoms trajectories were added to the models. Successful CBT for anxiety in children is associated with less depression symptoms for as long as four years. Anxiety symptom improvement appears to be a stronger predictor that demographic variables and parent mental health.

Cognitive behavioral therapy (CBT) is a well-established treatment for youth anxiety disorders, with solid meta-analytic evidence of its’ efficacy and effectiveness (James et al., 2020; Wergeland et al., 2021). However, youth anxiety disorders are associated with risk of multiple other mental health problems that may occur simultaneously or arise later as a consequence of living with anxiety. The most common mental health domain associated with anxiety is depression symptoms (Cummings et al., 2014). However, knowledge of the effects on depression symptoms after cognitive behavioral therapy (CBT) for youth anxiety disorders are scant (James et al., 2020). The focus of the current report is therefore on depression symptom trajectories following CBT for anxiety disorders in youth.

Multiple conceptual frameworks exist that can help shed light on the anxiety-depression overlap. Theories that try to explain the overlap focus on biological, psychological, or social dimensions. Biological theories have considered noradrenalin deficits and other endocrinological factors, sleep, aspects of neurotransmission, and/or genetics (e.g., Herane-Vives et al., 2018; Kraus et al., 2017). Psychological theories focus on attachment and other relations, self-control, cognitions and behaviors, and/or stress dimensions (e.g., Cummings & Cicchetti, 1990; Milrod et al., 2014). Attachment theory focuses on how early experiences of unavailable or inconsistent primary caregivers makes an individual vulnerable to feeling unworthy of being loved or cared for, which again is linked with experiencing loss, low self-esteem, rejection, and stress (Bowlby, 1973; Reinecke & Simons, 2005). Another set of conceptual frameworks focus on socio-cultural factors (e.g., Lorenzo-Blanco et al., 2012). The socio-cultural factors that have been proposed to be linked to depression include the immediate family environment with aspects such as parenting dimensions (e.g., warmth, consistency, communication) that are empirically linked to depressive symptomatology in children (see Goodman et al., 2020, for review). Socio-cultural models also include the wider meso- and macrosystem focusing on issues such as economic downfall being associated with more severe depression and suicide (Reeves et al., 2014) and social media use being linked to depression among youth (Bozzola et al., 2022).

Alongside biological, psychological, and socio-cultural theories that try to explain the anxiety-depression overlap, other conceptual frameworks focus on pathways, i.e., how anxiety can lead to depression and/or how depression can lead to anxiety. The Development Path Model (Cummings et al., 2014) proposed three different developmental pathways underlying the anxiety-depression disorder comorbidity. A first developmental pathway (pathway “A”) represents anxiety and depression as separate but related disorders (Affrunti & Woodruff-Borden, 2014; Cummings et al., 2014). This pathway concerns children with a genetic vulnerability to anxiety, for whom anxiety is argued to be the primary disorder, whereas comorbid depression contributes to negatively influence these children’s functioning. When the anxiety is not treated, stress from the anxiety can become a risk factor for comorbid depression (Rice et al., 2017). A second developmental pathway (pathway “B”) concerns children with genetic vulnerability to both anxiety and depression (Cummings et al., 2014). In this case, anxiety and depression disorders are considered co-primary. A third developmental pathway involves a genetic vulnerability to depression, where stress from depression can become a risk factor for the development of anxiety (Jacobson & Newman, 2017).

The models that have been proposed to explain depression etiology and maintenance have also influenced the conceptual frameworks that in turn guide treatment principles for depression. The current study focuses on CBT, which is built on cognitive and behavioral theories for how to understand depression origins and maintenance factors, with particular emphasis on cognitive theories of learned helplessness (e.g., Abramson et al., 1978; Beck, 1987) and behavioral learning theories (e.g., Lewinsohn, 1975). CBT centers on addressing and adjusting cognitions, for example attribution styles, which in depression is particularly associated with global, internal, and stable attributions being linked to experiences of failure, whereas attributions linked to experiences of success tend to be specific, external, and unstable (Bernaras et al., 2019). Behaviorally, CBT focuses on re-learning of action patterns and making behavior patterns more adaptable. Of particular relevance to the current study, a question that has hardly been addressed is the extent to which CBT principles that focus on anxiety (e.g., cognitive restructuring and exposure linked to anxiety-provoking situations) also influence depression factors (e.g. self-esteem, self-worth, everyday activity levels). There is a need to examine this over time, since the empirical evidence for the various proposed conceptual frameworks is limited, particularly in terms of studies that examine both prospective and co-occurring links between anxiety and depression over time, and outside university clinics.

The latest meta-analytic evidence for CBT anxiety trials rests on studies with outcome data up to 12 months post-treatment, with just one study (of 87) reporting outcomes at 24-months post-treatment in the latest Cochrane review (James et al., 2020). To address the need for long-term studies of depression effects from CBT anxiety trials, the current paper examines long-term effects of CBT on depression symptoms in youth up to four years after CBT for anxiety disorders. A few reviews have explicitly addressed long-term outcomes after CBT for anxiety disorders, from which some included studies have also considered depression outcomes. A narrative review found six studies that reported on depression symptoms up to 7 years post CBT for anxiety disorders in youth aged 7 to 17 years (*M* = 4.8 years; Gibby et al., 2017). Five of these six studies reported significant reduction in depression symptoms at long-term follow-up and one reported no change. In a meta-analysis of long-term CBT outcomes (> 2 years) across anxiety, depression, and post-traumatic stress disorders, Rith-Najarian and colleagues (2019) found an average effect size (Hedges’ *g*) of 2.2 for depression symptoms at long-term follow-up. However, this finding was based on only two studies. The most recent narrative review of CBT outcomes found reduced depression after CBT for youth anxiety disorders in four of 11 studies that had examined this (Luo & McAloon, 2021).

There are additional studies examining depression outcomes following CBT for anxiety disorders in youth that were not included in Luo and McAloon’s (2021) review. A study with 80 youths aged between 11 and 17 years with anxiety disorders examined the youths’ depressive symptoms two years after CBT for anxiety (Silk et al., 2019). The results showed that the youth who had responded immediately after CBT had lower levels of depressive symptoms at two years post CBT compared to non-responders. An important feature of this trial was the ability to show specific effects on depression based on CBT response status. These are promising findings, but the Silk et al. (2019) trial was conducted in a university clinic setting and results may not be generalizable to community settings.

Another study examined depression outcomes in a naturalistic follow-up of the Child/Adolescent Anxiety Multimodal Study (CAMS), a large multi-site trial examining CBT and other evidence-based treatment programs for youth anxiety. The study showed that treatment response (defined as much or very much improved on the Clinical Global Impressions-Improvement Scale) and remission (defined as loss of all study entry anxiety diagnoses) predicted a low depression trajectory 3 to 11 years post treatment among 319 youths aged 10–26 years (Keeton et al., 2019). Thus, this important trial showed promising findings for depression outcomes following evidence-based anxiety treatment. However, CAMS was conducted across six top-ranked university sites and did not specifically examine the effects of CBT, which may limit generalizability to CBT conducted in community clinics.

The current study examines data up to four years post-treatment from a randomized controlled trial (RCT) comparing individual and group CBT versus waitlist in a routine clinical care setting (i.e., an effectiveness study). The RCT findings showed that both CBT arms outperformed waitlist, with no significant differences between individual and group CBT in diagnostic recovery, anxiety symptoms, or depression symptoms (Wergeland et al., 2014). Previous long-term studies from this RCT have shown further improvement after four years on diagnostic recovery, anxiety symptoms, and depression symptoms (Kodal et al., 2018). In the current article, we provide new analyses of the data on the important issue of predictors of depression over the four-year assessment period of the trial. We focused on two potential pathways linking anxiety to depression trajectories following CBT for anxiety disorders. First, we examined if full diagnostic recovery from anxiety disorders at post-CBT was associated with less depression symptoms over time. This is conceptually relevant as it helps shed light on the potential developmental pathways between anxiety disorders and subsequent depression (Pathway “A”; Cummings et al., 2014). Second, we examined if the anxiety symptom trajectories from baseline across post-CBT and follow-ups one and four years later were associated with the depression symptom trajectories across the same time-period. This is conceptually relevant as it helps shed light on the proposed co-occurrence between anxiety and depression presentations (Pathways “A” and “B”; Cummings et al., 2014). Thus, focusing on both diagnostic recovery and symptom trajectories may provide the field with important empirical documentation of both a prospective and a co-occurring anxiety and depression link.

When examining these unresolved issues concerning anxiety and depression, it is also important to consider potential additional predictors and/or confounders. A handful of studies have examined predictors of long-term effects on depression following CBT for youth anxiety disorders. The only predictor found in more than one study was the initial trial outcome, i.e., the post-treatment result (see Gibby et al., 2017, for review). The RCT that the current data are from has examined predictors of anxiety diagnostic recovery and anxiety symptom reduction at four years follow-up. The findings were that lower family social class, poorer treatment motivation, and a primary disorder of social anxiety disorder were associated with poorer long-term outcomes (Kodal et al., 2018). However, predictors of the depression symptoms at four years follow-up and/or predictors of the depression trajectories over time in this RCT have not previously been examined.

In the current study, we considered multiple potential predictors when examining the long-term depression outcomes after CBT for youth anxiety disorders that were informed by the conceptual framework outlined above. In terms of background factors, we included youth age and gender because females and older youth tend to show more internalizing symptoms than males and younger youth (Rognstad et al., 2022; Solmi et al., 2023). We also included family social class because poor socio-economic status is a risk factor for mental health problems (Bøe et al., 2021). Finally, we included parent mental health. In light of the conceptual frameworks outlined above, parent mental health can influence anxiety and depression both via biological, psychological, and socio-cultural factors. Empirically, studies have shown that higher mental health symptoms in parents are associated with both higher parent-reported youth and youth self-reported mental health symptoms in youth (Fjermestad et al., 2017; Möller et al., 2016). Transactional studies indicate that over time, parent mental health may also influence how youth depression develops (e.g., Silverman et al., 2021). Importantly, youth internalizing problems also influence parent mental health (Fanti et al., 2013), so including parental mental health in longitudinal models is important.

The first research question is: *Does recovery from all inclusion anxiety disorders at post-treatment predict depression symptom trajectories from pre-treatment and up to four years post-treatment?* The second research question is; *Does change in anxiety symptoms predict change in depression symptoms from pre-treatment and up to four years post-treatment?* In line with meta-analytic and narrative review evidence, we hypothesized that initial recovery, defined as loss of all anxiety diagnoses, would predict a declining depression symptom trajectory up to four-year post-treatment, and that anxiety symptom change would predict depression symptom change across the time span from pre-treatment to four-years follow-up (Gibby et al., 2017; Luo & McAllon, 2021; Rith-Najarian et al., 2019).

## Methods

### Sample and Procedures

The sample comprised 179 youth aged 8 to 15 years at baseline (*M* = 11.5 years, *SD* = 2.1; 52.5% boys; 47.5% girls) who participated in a comparative randomized controlled trial (RCT) of individual CBT, group CBT, and 12-week waitlist. At four-year follow-up, the mean age was 15.5 years (range 11 to 21). The primary inclusion criterion was a DSM-IV (American Psychological Association, 2000) primary diagnosis of social phobia (SOP; 46.4%), separation anxiety disorder (SAD; 32.6%), and/or generalized anxiety disorder (GAD; 21.0%). All youth were regular referrals to one of seven outpatient community mental health clinics. Study exclusion criteria were psychotic disorders, intellectual disability, or pervasive developmental disorder. Depression was not an exclusion criterion, but the primary diagnosis had to be an anxiety disorder. See Table 1 for demographic background for the sample, divided by those who participated and those who did not participate in the long-term follow up.

**Table 1:** Sample characteristics

Variable	Participated at follow-up (n = 139)	Did not participate at follow-up (n = 40)
Gender		
Female	94 (67.6%)	24 (60.0%)
Male	45 (32.4%)	16 (40.0%)
Single parent family	25 (17.9%)	11 (28.2%)
Ethnicity		
European-White	128 (92.1%)	33 (82.5%)
Asian	3 (2.2%)	0 (0.0%)
Not reported	8 (5.7%)	7 (17.5%)
Family Social Class		
Low	15 (10.8%)	2 (5.0%)
Medium	44 (31.7%)	11 (27.5%)
High	68 (48.9%)	15 (37.5%)
Not reported	12 (8.6%)	12 (30.0%)
Principal anxiety diagnosis		
SOP	64 (46.0%)	20 (50.0%)
SAD	49 (35.3%)	9 (22.5%)
GAD	26 (18.7%)	11 (27.5%)
M age at follow-up	15.4 years (SD = 2.5)	16.2 years (SD = 2.1)

All derived based on the Anxiety Disorders Interview Schedule (ADIS)

*SOP* Social phobia, *SAD* Separation anxiety disorder, *GAD* Generalized anxiety disorder

The youth were randomized to individual CBT (*n* = 77), group CBT (*n* = 67), or 12-week waitlist (*n* = 37). After waitlist, the latter 37 youth were re-randomized to either individual (*n* = 16) or group CBT (*n* = 21). The present study focuses on anxiety and depression symptom trajectories across four timepoints (pre-, post-, one-, and four-years post-CBT). The study was approved by the review board for ethics in medical health research (REC-West). Before participation, parents provided written consent on behalf of themselves and the participating youth and children above 12 years provided verbal assent. A gift card of USD 50 was offered participants who participated in the four-year follow-up.

### Treatment and Therapists

The CBT program was the 4th edition of the FRIENDS for life manual (Barrett, 2004, 2008). This is a 10-session program followed by two booster sessions one and two months post-treatment. Therapists included ten psychologists, six special educators and one social worker, working as regular clinic employees, with between 3 and 27 years of clinical experience between them. Therapists delivered the CBT program with acceptable adherence (M = 4.79; SD = 0.75) and competence (M = 4.39; SD = 0.70), both measured on a 0 (poor) to 6 (excellent) range on the Competence and Adherence Scale for Cognitive and Behavioral Therapy CAS-CBT (Bjaastad et al., 2016).

### Instruments

**Anxiety Disorders Interview Schedule for DSM-IV, child and parent versions** (ADIS-IV C/P; Silverman & Albano, 1996) were used to determine diagnosis and clinical severity ratings (scale 0–8) for included anxiety diagnoses (i.e., SOP, SAD and GAD). Interrater agreement for the presence of an inclusion anxiety diagnosis was good (κ = 0.84 for ADIS-C, κ = 0.86 for ADIS-P), based on masked re-rating of 20% of video-recorded interviews.

**The Registrar General Social Class Coding Scheme** (Currie et al., 2008) was used to measure family social class. This scheme classifies parent occupational status into five rank ordered socio-economic status classes. Family social class was defined by the highest ranking parent in the family.

**Spence Children’s Anxiety Scale, child and parent versions** (SCAS-C/P, Spence, 1998) were used to assess youth anxiety symptoms. The SCAS comprises 38 items reported on a 4-point Likert scale. Internal consistency was α = 0.88 for youth and α = 0.81 for parents.

**Short Mood and Feeling Questionnaire**, **child and parent versions** (SMFQ, Wood et al., 1995) were used to measure youth depression symptoms. The SMFQ comprises 13 items reported on a 3-point Likert scale. Internal consistency was α = 0.86 for youth and α = 0.88 for parents.

**Depression Anxiety Stress Scale** (DASS, Lovibond & Lovibond, 1995) was used to measure parent mental health. The DASS comprises 21 items reported on a 4-point Likert Scale. Internal consistency was α = 0.95 for parents.

### Data Analytic Plan

Because about one half of the sample were treated in groups, the data were partially clustered. The cluster-level variance for post-treatment youth-reported depression ranged from 0.01 to 0.11. It was determined that the clustering effects were too low to affect the overall models (Guo, 2005). In the growth curve models, missing data were handled using full information maximum likelihood. In all analyses, data from the ICBT and GCBT conditions were combined, as analyses in the main trial showed no difference between the two formats (Wergeland et al., 2014; Kodal et al., 2018).

To examine research question 1, the difference between diagnostically recovered and non-recovered youth (i.e., those who had lost all anxiety diagnoses versus those who had not) were run as regression analyses in SPSS version 28 with full recovery as the grouping variable and the SMFQ trajectories for youth and parents, respectively, as dependent variables. A *p*-value of 0.05 was used as cutoff to determine if the difference between the two means were statistically significant. To examine research question 2, we ran growth curve analyses as four regression models predicting the depression symptom trajectories from pre-treatment to 4-years follow-up using the lmer package in R version 12.0 (Bates et al., 2023). In these analyses, each participant’s anxiety and depression scores for the four timepoints (pre-, post-, one-, and four-years post-CBT) were restructured to represent the respective symptom trajectories, matched with a time variable ranging from 0 to 4 representing the four measurement points. Two models were conducted for within-informant effects (youth-reported anxiety symptom trajectory predicting youth-reported depression symptom trajectory, and parent-reported anxiety symptom trajectory predicting parent-reported depression symptom trajectory). Then, two models were run for cross-informant effects (the youth-reported anxiety symptom trajectory predicting the parent-reported depression symptom trajectory, and the parent-reported anxiety symptom trajectory predicting the youth-reported depression symptom trajectory).

All four models were conducted in three steps, (a) with random intercept, and slope (time), age, gender, and family social class as fixed predictors, (b) adding the anxiety symptoms trajectory (reported by youth and parents in different models) as a time-varying predictor, and (c) adding parent mental health at baseline as a fixed-time predictor. Model fit was assessed based on comparison of the Akaike Information Criterion (AIC; Cavanaugh & Neath, 2019) across models. Lower AIC values indicate better model fit, and overfitting is indicated if the AIC values are not reduced when more predictors are added. The amount of explained variance (*adjusted R*^*2*^) per outcome was also calculated for each step.

## Results

Data were available for 80.9% of the original sample at one-year follow-up and for 88.4% at 4 years follow-up. Analyses showed that for parent reports, youth who lost all their inclusion anxiety diagnoses at post-treatment (i.e., recovered) had a significantly lower parent-reported mean depression symptom trajectory (i.e., lower levels of depression symptoms over time) up to four years post-treatment, compared with youth who did not lose all diagnoses (*M* SMFQ-P_recovered_ = 3.84 (*SD* = 3.95) versus M SMFQ-P_non−recovered_ 6.28 (*SD* = 5.31), *p* < 0.001, *d* = − 0.49). The difference was not significant for youth self-report (M SMFQ-C_recovered_ = 5.25 (*SD* = 5.24) versus M SMFQ-C_non−recovered_ 6.32 (*SD* = 5.79), *p* = 0.052, *d* = − 0.19).

The within-informant effects for the depression symptom trajectory are shown in Table 2. For the youth-self-reported depression trajectory, the first model (Model 1a) with time, age, gender, and family social class as predictors, showed youth-self-reported depression decreased over time, with higher youth age, female gender, and lower family social class predicting less depression symptom change (i.e., higher levels of depression symptoms over time). When the youth-self-reported anxiety trajectory was added (Model 1b), this explained variance in the youth-self-reported depression trajectory above and beyond time, gender, and family social class. This means that if youth-reported anxiety symptoms had increased over time, so had youth-reported depression symptoms. Higher youth age remained a significant predictor of less depression change (i.e., more depression symptoms). When parent-self-reported mental health was added to the model (Model 1c), this was not a significant predictor.

**Table 2:** Parameter estimates for the growth curve models, within-informant effects

	Model a		Model b		Model c	
	Est	95% CI	Est	95% CI	Est	95% CI
**SMFQ youth**						
Fixed effects						
Intercept	-1.31	-5.38,2.76	**-4.17**	-7.39,-0.95	**-3.91**	-7.18,-0.64
Slope	**-0.60**	-0.94,-0.27	0.14	-0.16,0.44	0.08	-0.24,0.40
Age	**0.36**	0.05,0.67	**0.27**	0.03,0.52	**0.27**	0.02,0.51
Gender	**2.23**	0.92,3.54	0.85	-0.19,1.90	0.81	-0.24,1.87
Social class	**1.11**	0.04,2.18	0.29	-0.56,1.14	0.25	-0.60,1.08
SCAS youth			**0.20**	0.17,0.22	**0.19**	0.17,0.22
DASS parent					0.00	-0.00,0.00
Model fit						
AIC	3283.167		3070.731		3007.061	
R^2^	0.09		0.39		0.39	
**SMFQ parent**						
Fixed effects						
Intercept	**4.58**	0.98,8.18	-1.78	-5.09,1.54	-1.71	-5.00,1.59
Slope	**-1.02**	-1.32,-0.72	-0.07	-0.36,0.22	-0.09	-0.39,0.20
Age	0.16	-0.12,0.44	0.18	-0.06,0.43	0.17	-0.07,0.41
Gender	0.14	-1.03,1.30	-0.02	-1.04,1.01	0.03	-0.99,1.04
Social class	0.59	-0.36,1.54	-0.11	-0.96,0.73	-0.09	-0.93,0.75
SCAS parent			**0.20**	0.17,0.23	**0.20**	0.17,0.23
DASS parent					0.00	-0.00,0.00
Model fit						
AIC	3227.121		3014.057		2941.743	
R^2^	0.06		0.31		0.31	

Significant estimates in bold. R2 = marginal (i.e., only fixed effects). Model a = time (i.e., slope) and demographic characteristics (i.e., age, gender, and social class as predictors). Model b = anxiety symptom trajectory (i.e., SCAS) added. Model c = Parent mental health (i.e., DASS) added

*Est* Estimates, *CI* confidence intervals (with lower and upper bound)

For the parent-reported youth depression trajectory, the first model (Model 1a), with time, age, gender, and family social class as predictors showed parent-reported youth depression decreased over time, regardless of youth age, gender, and family social class. When the parent-reported youth anxiety trajectory was added (Model 1b), this explained variance in the parent-reported youth depression trajectory above and beyond time. This means that if parent-reported youth anxiety symptoms had increased over time, so had parent-reported youth depression symptoms. When parent-self-reported mental health was added in the final model (Model 1c), this was not a significant predictor of the parent-reported youth depression trajectory alongside the parent-reported youth anxiety trajectory. The AIC values indicated best fit for the models that included all the predictors (i.e., time, age, gender, family social class, anxiety symptoms, and parental mental health), both for youth and parent reported youth depression outcomes.

The cross-informant effects for the youth depression symptom trajectory are shown in Table 3. When the parent-reported youth anxiety trajectory was added to the model predicting the youth-self-reported depression trajectory (Model 2b), this was a significant predictor. Youth age and gender were also significant predictors, with higher parent-reported youth anxiety reduction predicting higher youth-self-reported depression reduction, and higher youth age and female gender predicting less depression reduction. When parent-self-reported mental health was added to the model (Model 2c), this was a significant predictor of the youth-self-reported depression trajectory alongside youth age and gender, and parent-reported anxiety symptom trajectories for youth. This model (2c) showed that more parental mental health problems at pre-treatment was associated with more youth-reported depression symptoms over time. Family social class was not a significant predictor in these models.

**Table 3:** Parameter estimates for the growth curve models, cross-informant effects

	Model a		Model b		Model c	
	Est	95% CI	Est	95% CI	Est	95% CI
**SMFQ youth**						
Fixed effects						
Intercept	-1.31	-5.38,2.76	**-4.68**	-8.79,-0.58	**-4.61**	-8.76,-0.47
Slope	**-0.61**	-0.94,-0.27	-0.08	-0.46,0.29	-0.14	-0.52,0.24
Age	**0.36**	0.05,0.67	**0.35**	0.05,0.65	**0.36**	0.05,0.66
Gender	**2.23**	0.92,3.54	**2.14**	0.88,3.40	**2.12**	0.85,3.39
Social class	**1.11**	0.04,2.18	0.78	-0.26,1.82	0.75	-0.31,1.80
SCAS parent			**0.11**	0.08,0.15	**0.11**	0.07,0.15
DASS parent					**0.01**	0.00,0.01
Model fit						
AIC	3283.167		3118.511		3043.002	
R^2^	0.09		0.15		0.15	
**SMFQ parent**						
Fixed effects						
Intercept	**4.58**	0.98,8.18	**3.76**	0.16,7.36	**3.76**	0.17,7.34
Slope	**-1.02**	-1.32,-0.72	**-0.76**	-1.09,-0.44	**-0.79**	-1.12,-0.46
Age	0.16	-0.12,0.44	0.13	-0.14,0.40	0.12	-0.15,0.39
Gender	0.14	-1.03,1.30	-0.32	-1.47,0.84	-0.30	-1.46,0.85
Social class	0.59	-0.36,1.54	0.23	-0.72,1.17	0.23	-0.71,1.17
SCAS youth			**0.07**	0.04,0.09	**0.07**	0.04,0.09
DASS parent					0.01	-0.00,0.01
Model fit						
AIC	3227.121		3075.015		3004.234	
R^2^	0.06		0.10		0.11	

Significant estimates in bold. R2 = marginal (i.e., only fixed effects). Model a = time (i.e., slope) and demographic characteristics (i.e., age, gender, and social class as predictors). Model b = anxiety symptom trajectory (i.e., SCAS) added. Model c = Parent mental health (i.e., DASS) added

*Est* Estimates, *CI* confidence intervals (with lower and upper bound)

When the youth-self-reported anxiety trajectory was added to the model predicting the parent-reported youth depression trajectory (Model 2b), this was a significant predictor alongside time. This means that more youth-reported anxiety over time was associated with more parent-reported youth depression over time. When parent-self-reported mental health was added in the final model (Model 2c), this was not a significant predictor of the parent-reported youth depression trajectory. As for the within-informant models, the AIC values indicated best fit for the models that included all the predictors, both for youth and parent reported depression outcomes.

## Discussion

In this report from an RCT for youth anxiety disorders (original trial was (Wergeland et al., 2014), we found that the anxiety symptom trajectory predicted the depression symptom trajectory up to four years post-treatment. Across all the models we examined, the results showed that when anxiety symptoms had increased over time, so had depression symptoms. However, diagnostic recovery from anxiety disorders was only associated with less parent-reported depression symptoms (for youth) over time, but not with the level of youth self-reported depression symptoms. These findings are mainly in line with conceptual frameworks and empirical work (e.g., Cummings et al., 2014; Gibby et al., 2017; Keeton et al., 2019; Silk et al., 2019).

It is important to consider our findings in light of existing conceptual frameworks that have attempted to explain the anxiety-depression overlap in terms of etiology and pathways. One the one hand, it could be argued that the findings indicate stronger evidence for anxiety and depression symptom trajectories as overlapping, relative to anxiety recovery predicting later depression symptoms. Hence, arguably the empirical evidence presented herein are more consistently in line with the second developmental pathway for anxiety and depression (i.e. pathway “B”; co-occurring), and not in line with the first proposed pathway, i.e., pathway “A”, that primary anxiety leads to later depression (Cummings et al., 2014). On the other hand, however, the inconsistency between findings for youth self-report versus parent-report, weakens the argument for stronger evidence for one pathway versus another pathway, and more research is needed to empirically investigate anxiety-depression pathways over time.

It is important to consider why there was a contrast in the findings regarding diagnostic recovery and later parent report about youth depression, versus youth-self-reported depression An explanation could be that in the original RCT, there was no significant change in youth-reported depression from pre- to post-treatment, whereas there was a significant difference for parent-report about youth (Wergeland et al., 2014). Thus, from the youth perspective there may have been less depression change to predict. However, the differences in results should also be considered in light of informant discrepancies. Of particular relevance to the current study, youths and parents typically show low agreement in their ratings of levels of youth depression and anxiety symptoms (De Los Reyes et al., 2015; Rognli & Fjermestad, 2023). A reason for this discrepancy may rest on the fact that anxiety and depressive symptoms to a large degree are ”internal”, and thus not visible and therefor accessible for parents to assess (American Psychological Association, 2000). Also, adolescents and parents may interpret the questions differently, and may have varying thresholds for defining a symptom or behavior as problematic (Kramer et al., 2004). As found in the current study, within-informant associations (e.g. youth-reported anxiety and youth-reported depression) are usually higher than cross-informant associations (e.g., youth-reported anxiety and parent-reported depression), particularly for internalizing problems (De Los Reyes et al., 2015). This highlights the importance of examining both perspectives. Finally, parents are usually the initiators of treatment for youth, so parent assessments are highly relevant in terms of assessing youth’s clinical needs. The finding is a reminder to consider multiple informants also in terms of longer-term outcomes.

In terms of overlap with previous empirical studies, most extant follow-up studies have had shorter time intervals between treatment and follow-up (Gibby et al., 2017). Our findings contribute to the growing body of evidence that anxiety recovery is associated with long-term depression symptom outcomes. Furthermore, most previous studies have been conducted in university clinics, so the current study provides evidence of long-term predictors of depression outcomes when CBT is delivered in community clinics.

Older youth age, female gender, lower family social class, and poorer parent mental health negatively influenced the depression symptom trajectory in some of the models. This means that older age, being female, and coming from a lower family social class were all associated with higher levels of depression over time. Importantly, these effects never went beyond the effect of the anxiety symptom trajectory, meaning that higher anxiety levels over time is the main predictor of higher depression levels over time. Nevertheless, all these factors (i.e., age, gender, social class) have previously been found to pose a risk for depression (e.g., Ghandour et al., 2019; Rognli & Fjermestad, 2023). Although these variables did not predict depression above anxiety improvement, these findings indicate that older youth, girls, families with lower social class, and those whose parents have mental health problems may be at particular risk and in need of CBT that also addresses depression.

It is important to consider the study’s findings in light of the youths’ age. The peak age of first being diagnosed with any anxiety disorder is 5.5 years, with 51.8% of lifetime anxiety disorders estimated to have their onset before age 18 years (Solmi et al., 2022). Note that the low age of onset reflects the inclusion of separation anxiety disorders, which typically occurs much earlier than the other anxiety diagnoses represented in the current sample. Social phobia has 14.5 years as the peak onset age with generalized anxiety disorder one year later at 15.5 years (Solmi et al., 2022). Depression, on the other hand, has 19.5 years as the peak age of onset and only 13.2% of lifetime patients are estimated to have their onset before age 18 years (Solmi et al., 2022). Ideally, we should therefore have followed our participants further into young adulthood. It is nevertheless important to bear in mind that we have examined predictors of depressive symptoms, not disorders.

The strengths of the current study include a relatively large sample followed up until four years post-treatment with little dropout at the follow-up times, adding to the power of the analyses and thus the ability to detect smaller associations. We recruited the sample from community clinics and used well-validated multi-informant diagnostic instruments and symptom measures. An original feature of the current study is that we considered cross-informant effects. Specifically, we explored if youth-reported data influenced parent-reported data and if parent-reported data influenced youth-reported data.

The study also has limitations. Most importantly, the lack of a control group beyond the post-treatment measurement point makes it challenging to causally infer that CBT had an effect on later depression. We also have limited overview over life events, including psychological treatments received, in the period from post-treatment to four-years follow-up. Albeit the sample size is decent for an effectiveness long-term follow-up study, in terms of statistical power it only allowed us to examine a handful of predictors. Social phobia is consistently associated with poorer treatment response relative to other anxiety disorders (Wergeland et al., 2021). Thus, the fact that almost half our sample had social phobia as their primary anxiety disorder may have substantially influenced the results. The majority of our sample were European-White and the findings may not be generalizable to other ethnic groups. Because the Norwegian language does not distinguish between (birth assigned) sex and (social) gender, we also cannot be specific on which of these dimensions (sex versus gender) that our gender-labelled variable reflects for each participant. It is also important to see the findings in light of the CBT program used. The FRIENDS program does contain some general coping skills elements and a self-concept section, that may have been particularly relevant for depression outcomes, albeit we cannot check this with data since all participants received the same manual. The use of other manuals may have led to different results.

### Implications and Concluding Remarks

Our findings that CBT for anxiety has long-lasting effects on depression is promising and provides further support to the multitude of evidence for CBT effects. Clinicians can convey an expectation of enduring effects at the end of treatment to patients. Successful treatment of anxiety seems to prevent the development of later depression symptoms. Due to the common overlap between anxiety and depression, it can be relevant to assess both at treatment onset and later evaluation/decision points. It is important to note, however, that our sample had a primary anxiety disorder, and patients with co-morbid anxiety and depression may need CBT approaches that are better tailored for both (Martinsen et al., 2021; Weersing et al., 2017). As indicated by transdiagnostic approaches, several cognitive-behavioral tasks such as problem solving, activity planning, emotion recognition and regulation tasks, as well as cognitive restructuring can be applied to both anxiety and depression problems. We examined demographic variables (youth age and gender and family social class) as well as parent mental health as potential confounders of the anxiety-depression associations, with the finding that none of these “outpowered” the role of anxiety trajectories for depression outcomes.

Nevertheless, it is important to note that the models that included more predictors showed better model fit. Since this is expected up until potential overfitting (Stoltzfus, 2011) careful consideration of what predictors should be included is needed in future studies. Albeit our chosen demographic factors (age and gender) failed to explain variance, we could have focused on other potential confounders and predictors. It would be particularly useful to the field to be informed by conceptual frameworks and examine confounders believed to influence the co-occurrence of anxiety and depression, such as genetics, attachment, and social functioning (e.g., Herane-Vives et al., 2018; Milrod et al., 2014). In terms of genetics, the number of studies on the role of various genetic origins for anxiety disorders are rapidly increasing. Albeit specific genes have yet to be consistently identified, recent reviews of the genetics of anxiety disorders recommend including genetics and biomarkers as predictors in treatment studies (Baba et al., 2022; Tomasi et al., 2019). In terms of attachment and social functioning, measures range from observation paradigms to parent and self-report measures of both direct attachment but also related concepts such as emotional relations and/or communication. Examples of well-validated attachment and social relationship measures that future studies could consider as predictors include the Inventory of Parent and Peer Attachment (Armsden & Greenberg, 1987) and the Experiences in Close Relationships-Revised Child Version (Brenning et al., 2011). Future studies should also include multiple assessment points and also control for post-treatment variables like additional treatment. We conclude that CBT for anxiety contributes to fewer symptoms of depression over time.

## Data Availability

Data are available upon request to the first author.
